# A New Era of Research in the U.S. Department of War Serum Repository: A Review of Published Studies, 2013–2024

**Published:** 2026-09-20

**Authors:** Sithembile L. Mabila, Scott J. Russell, Adewumi Adegboye, Shauna L. Stahlman

**Affiliations:** Epidemiology and Analysis Branch, Armed Forces Health Surveillance Division, Public Health Directorate, Defense Health Agency, Silver Spring, MD

## Abstract

The Department of War Serum Repository (DOWSR, formerly DODSR ^1^ ) was established in 1989 after the initiation in 1985 of the U.S. Department of War’s universal mandatory human immunodeficiency virus (HIV) screening program. DOWSR, which is currently maintained by the Armed Forces Health Surveillance Division (AFHSD), preserves over 78 million serum specimens. Similar to a study that investigated research conducted from 1985 through 2012 utilizing the serum repository, this study evaluated DOWSR specimen availability, stratified by demographic variables, and conducted an analysis to determine total number and types of research studies performed using the DOWSR from 2013 through 2024. Two methods were employed: a structured PubMed search and an exhaustive online search based on records of requests for serum samples from AFHSD. Results show that the focus of DOWSR’s research within the past decade has increased, especially in regulatory molecules, autoimmune indicators, and microRNA expression patterns, with increases in both the number of sponsored studies and variety of analytes investigated. Changes in the types of studies pursued by DOWSR research may reflect changing trends in funded research regarding health concerns for service members and veterans.

**What are the new findings?**

This review identified 112 published and 21 non-published studies that used DOWSR sera during the study period. Of the published 112 studies, 30 investigated infectious diseases, 25 investigated autoimmune disorders, 13 investigated neoplasms, and 11 investigated chronic metabolic outcomes. More studies using DOWSR specimens were published on environmental exposures and autoimmune disorders compared to prior years.

**What is the impact on readiness and force health protection?**

To inform risk assessments and validate force health protection measures, researchers and public health professionals use the DOWSR to retrospectively analyze emergent pathogens, exposures, and unrecognized health threats. This medical surveillance and research each enhances military readiness by improving understanding of debilitating conditions that affect service members, such as autoimmune and infectious diseases, neoplasms, and chronic metabolic conditions.

The U.S. Department of War Serum Repository (DOWSR) is an important biomedical resource, with millions of serological specimens collected from military personnel over multiple decades. The DOWSR is believed to be the largest bank of human serum in the world. The DOWSR was established in 1989 after the beginning of the Department of War (DOW)’s universal mandatory human immunodeficiency virus (HIV) screening program, initiated in 1985. ^2 - 6^ The DOWSR’s initial purpose was to store residual serum samples to study and refine HIV testing, establish epidemiological risk factors, and investigate other infectious diseases. ^2 , 3^ Over time, its mission expanded significantly, to include collection and storage of operational deployment specimens in addition to those from all branches of service of the U.S. Armed Forces. ^2 , 4 , 5^ The policies for serum sample collection in the armed forces have not changed since the last DOWSR review study. ^2^

The DOWSR is currently maintained by the Armed Forces Health Surveillance Division (AFHSD), preserving over 78 million serum specimens. ^2 , 3^ Approximately 1-2 million specimens are added annually. ^5^ One of the most important components of the DOWSR’s utility is its direct link to the Defense Medical Surveillance System (DMSS), which allows researchers and public health investigators to link specific serum specimens to an individual’s service history, deployments, and health outcomes. ^7^ This longitudinal data link makes the DOWSR an invaluable epidemiological surveillance tool, supporting force health protection, disease prevention, and public health policy.

Prior studies have investigated the origination, utility, and description of the DOWSR. ^2 , 3 , 7 , 8^ The main objective of this study is to update and compare 2013-2024 data and results to the previously published description, utility, and review of published studies using the DOWSR from 1985 through 2012. ^2^ This report presents the total number and types of research studies performed using the DOWSR from 2013 through 2024, with an update on the number of specimens currently available.

## Methods

Data on serum specimens through the end of 2024 were obtained from DMSS. Serum availability was stratified by sex, age, and branch of service. Serum samples were not accessed; only data on the availability of specimens were utilized.

To determine the total number of publications using DOWSR serum samples, the authors conducted a review of internal, controlled-access AFHSD records, and of external public records. First, external publicly accessible scientific literature in the PubMed database was queried using the following text string: “department of defense biorepository”, “dod biorepository”, “DoDSR”, “dodsr”, “dod serum repository”, “dod sr”, “Department of Defense Serum Repository (DoDSR)”, “Armed Forces Health Surveillance Center” (and “Branch” and “Division”), “Department of Defense Serum Repository”, “military”, “dod”, “defense” and “biorepository*”, “repositor*”, “ stored”, “storage”, “bank”, and “frozen”—in English. There were 349 publications initially identified, which the researchers then restricted by the additional criteria: research studies conducted by or for the U.S. military as identified by known authors, sponsors, and institutions, confirmed use of DOWSR samples in the paper’s methodology, and publication status within the study period, 2013-2024. 100 of the 349 externally identified studies met all inclusion criteria **(Figure 1)** .

**FIGURE 1.**
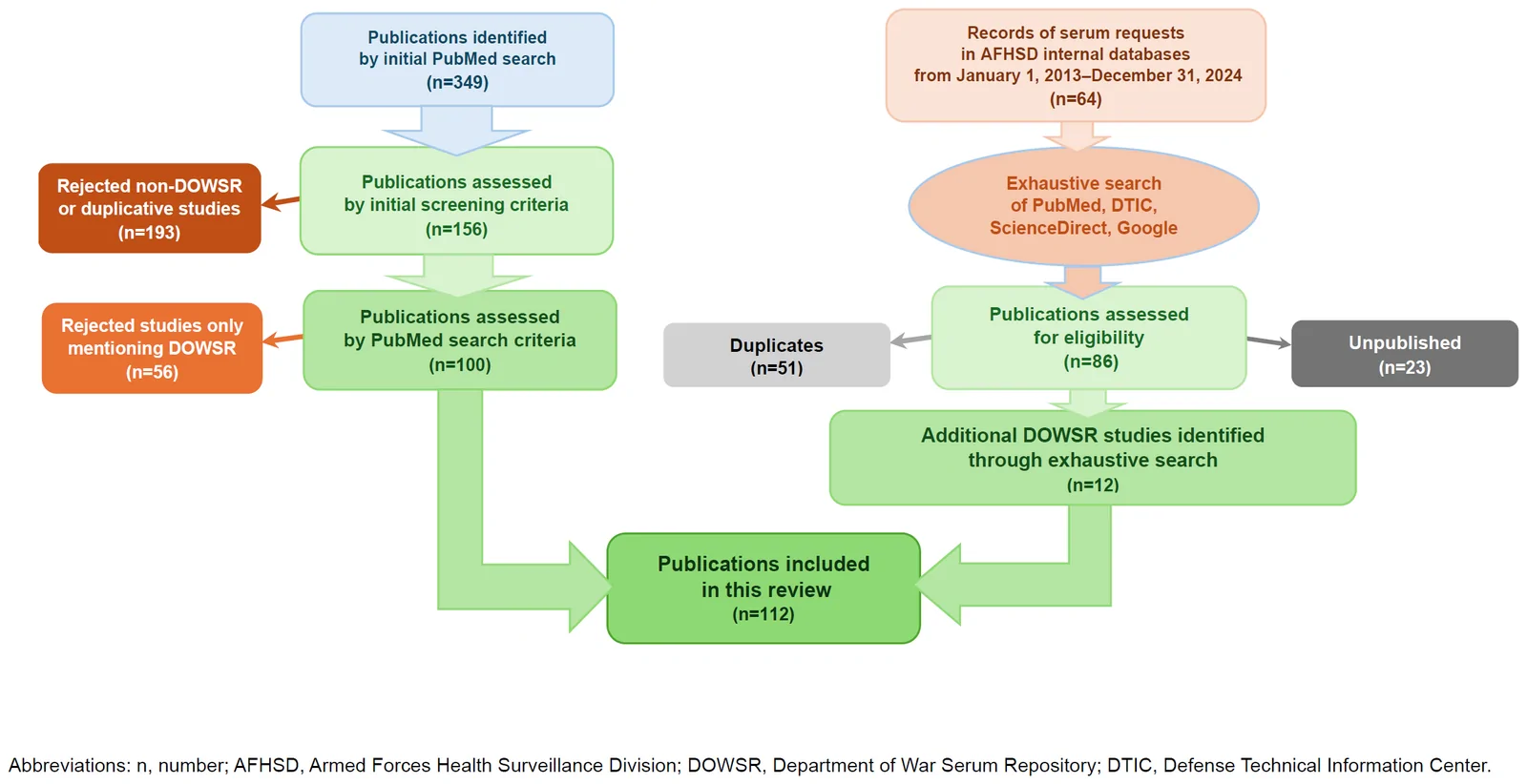
Department of War Serum Repository Results in Literature Search Using Both Alogrithmic and Intense Methods

The second part of the analysis was an internal search utilizing a list of serum-based studies queried at AFHSD that were supported from 2013 through 2024. The DOWSR management database tool and research protocol review tracker are record management systems implemented to manage and track all research and serum studies supported by AFHSD. According to their records, 164 unique serum requests were completed and provided to their requesters during the study period.

Additionally, for each DOWSR request, the names of the investigators and health condition(s) under study were searched first in PubMed, then if not found, in Google, then, finally, in the Defense Technical Information Center (DTIC); DTIC is an organization within the U.S. Department of War (DOW) that serves as a central repository for scientific and technical records, including reports and both published and unpublished research. In each search, the full title, list of authors, then combination of partial title and author list were queried, with results reviewed for a match with internal records. This exhaustive search method identified an additional 12 articles that used DOWSR specimens and were published from 2013 through 2024.

These record searches resulted in a combined total of 112 published studies. A systematic literature review then determined the analyte(s) and health condition(s) investigated, methodology, and significance of findings. Neither a meta-analysis nor any other statistical analyses were performed, nor were there any *a priori* decisions for inclusion based on study design.

When possible, health outcomes were categorized based on the groupings defined in the previous study, ^2^ for ease of comparison, as was the case for 8 categories. Otherwise, categories were classified by the authors (for cases of burn pit exposure, osteoporosis, cardiovascular disease, tobacco use, deployment exposures; **(Table 4)** . This classification was based on the main outcome or, for studies without clear outcomes, exposure investigated. This approach was necessary chiefly to account for the emergence of exposure-focused studies, without defined outcomes, that did not conform to the previously established categories.

**TABLE 1.** Summary Statistics for Ages of Individuals at Serum Collections and Timings of Specimen Collections for Department of War Serum Repository ^a^

	Minimum	Maximum	Mean	Standard Deviation	Median
Age at date of collection, *y*	17	70	27.71	8.64	25
Specimens per person, *n*	1	35	5.52	4.74	4
Duration between specimens, *y*	0	34	1.40	1.32	1
Duration between first and last specimens, *y*	0	37	6.32	6.79	4

Abbreviation: *y* , years; *n* , number.

a Analysis restricted to samples with ages 17-70 years at collection.

**TABLE 2.** Counts of Individuals Who Provided Specimens to the Department of War Serum Depository and Numbers of Specimens per Individual

No.	No.	%
1	1,728,781	15.55
2	1,685,535	15.16
3	1,389,424	12.50
4	1,260,634	11.34
5	1,040,951	9.36
>5	4,011,106	36.08

Abbreviation: No., number.

**TABLE 3.** Analytes from Published Studies Utilizing Department of War Serum Depository Samples and Counts of Articles in Which Each Analyte Appears

Articles	Analytes Investigated
No.	
22	Regulatory molecules (cytokines, chemokines, hormones)
9	microRNA (miRNA) expression patterns
8	Rheumatoid arthritis factor; anti-cyclic citrullinated peptides, anti-bacterial serum antibodies
7	Cotinine levels
6	25-hydroxyvitamin D
5	Polycyclic aromatic hydrocarbons (PAHs)
4	Influenza A antibodies, C-reactive proteins
3	Multiple sclerosis (whole proteome autoantibodies, IgG systemic sclerosis autoantibodies); leishmaniasis; lupus nephritis anti-double stranded DNA antibodies; polybrominated biphenyls (PBBs); polychlorinated biphenyls (PCBs); human DNA
2	Crohn’s disease antibodies; SARS-CoV-2; irritable bowel disease; meningitis vaccine; tick-borne rickettsia; IgG for leptospirosis; polybrominated diphenyl ethers (PBDEs); Epstein-Barr virus (EBV) peptides; human papillomavirus (HPV); dengue virus; mumps virus; yellow fever; organochlorine pesticides; collagen, aggrecan synthesis biomarkers; bone turn-over biomarkers; adenovirus 36 antibody
1	Japanese encephalitis antibodies; granulomatosis with polyangiitis; IgG for *Neisseria gonorrhoeae* ; hemagglutinin pseudovirus anti-bodies; M-type phospholipase A2 receptor; herpes, syphilis antibodies; thrombospondin type-1 domain 7A (THSD7A); nutritional metabolites; immunoglobulin light chain amyloidosis; coccidioides IgG or IgM; *Amblyomma americanum* (lone star tick);, tick-borne ehrlichia; thyroid peroxidase (TPO) antibodies; B-cell follicular lymphoma (FL); diffuse large B-cell lymphoma (DLBCL); per-, polyfluoroalkyl substances (PFAS); protein biomarkers; inflammation-related candidate biomarkers: TNF receptor 2 (TNFR2), IL17A, VEGFR3, and resistin; lupus erythematosus interferon alpha; β-trace protein (BTP), β2-microglobulin (B2M); metanephrine (MN); normetanephrine (NMN); *Porphyromonas gingivalis* antibody; anti-salmonella, anti-norovirus antibodies; smallpox vaccine; IgG for *Borrelia burgdorferi* ; bacterial translocation biomarkers; soluble CD14 (sCD14); Ross River virus antibodies; hepatitis B antibody; hepatitis C antibody; monoclonal protein; free light chains; polychlorinated dibenzo-p-dioxins (PCDDs); polychlorinated dibenzofurans (PCDFs)

Abbreviations: miRNA, micro-ribonucleic acid; PAHs, Polycyclic aromatic hydrocarbons; IgG, immunoglobulin G; DNA, deoxyribonucleic acid; PBBs, polybrominated biphenyls; PCBs, polychlorinated biphenyls; SARS-CoV-2, severe acute respiratory syndrome coronavirus 2; PBDEs, polybrominated diphenyl ethers; EBV, Epstein-Barr virus; HPV, human papillomavirus; THSD7A, thrombospondin type-1 domain 7A; IgM, immunoglobulin M; TPO, thyroid peroxidase; FL, follicular lymphoma; DLBCL, diffuse large B-cell lymphoma; PFAS, per-, polyfluoro-alkyl substances; TNF, tumor necrosis factor; TNFR2, tumor necrosis factor receptor 2; IL17A, interleukin-17A; VEGFR3, vascular endothelial growth factor receptor 3; BTP, β-trace protein; B2M, β2-microglobulin; MN, metanephrine; NMN, normetanephrine; sCD14, soluble CD14; polychlorinated dibenzo-p-dioxins; PCDFs, polychlorinated dibenzofurans.

**TABLE 4.** Publication Counts from Studies Utilizing the Department of War Serum Repository, by Health Outcome and Analyte Investigated, 2013–2024

Health Exposure or Outcome	Antibodies	Regulatory Molecules, Biomarkers	Chemical Compounds	Human DNA	Nutrient	Total Analytes	Total Unique Studies
	No.	No.	No.	No.	No.	No.	No.
Infectious diseases	28	3		1		32	30
Neoplasms	3	9	3			15	13
Chronic metabolic	6	5	4		2	17	11
Autoimmune disorders	18	11	1	1	2	33	25
Non-specific	1	2	1			4	4
Vaccine-induced immunity	4				1	5	4
Physical injury ^a^		6			1	7	7
Mental illness		2	1			3	3
Burn pit exposure	1	6	4			11	8
Osteoporosis		2			2	4	3
Cardiovascular disease				1		1	1
Tobacco use		1				1	1
Deployment exposures ^b^		2	2			4	2
Total	61	49	16	3	8	137	112

Abbreviations: DNA, deoxyribonucleic acid; No., number.

a Includes studies on suicide.

b Non-specific deployment exposures.

## Results

### Contents of the DOWSR

At the end of this study’s surveillance period, December 31, 2024, the DOWSR contained just under a total of 78.6 million samples. The exclusion of unavailable samples and those without identifiable or demographic information resulted in 61.9 million samples from 11.4 million distinct service members. After the exclusion of samples with erroneous or outlier information (e.g., missing age, younger than age 17 years or older than age 70 years at collection, outlier SSNs with the top 0.01% serum sample counts, n=1,112), 61.3 million (78.1%) of the serum samples were available for researchers, from 11.1 million members, with an average age of 27.7 years (standard deviation [SD] 8.64, median 25) at time of serum collection. There was a mean of 5.5 samples per person (SD 4.75, median 4), with a mean of 1.4 years between sample collections (SD 1.32, median 1), and a mean range from first to last sample per person of 6.3 years (SD 6.79, median 4) **(Table 1)** .

Among the 61.3 million samples that met the inclusion criteria, contributors were mostly male (77.9%) and served in the Army (41.0)% (data not shown). The annual number of serum samples provided to the DOWSR steadily increased from 1985 to its peak in 2003 (n=2.3 million), then remained relatively stable until 2010, after which it slowly started to decrease to 1.3 million samples at the end of 2024 **(Figure 2)** .

**FIGURE 2.**
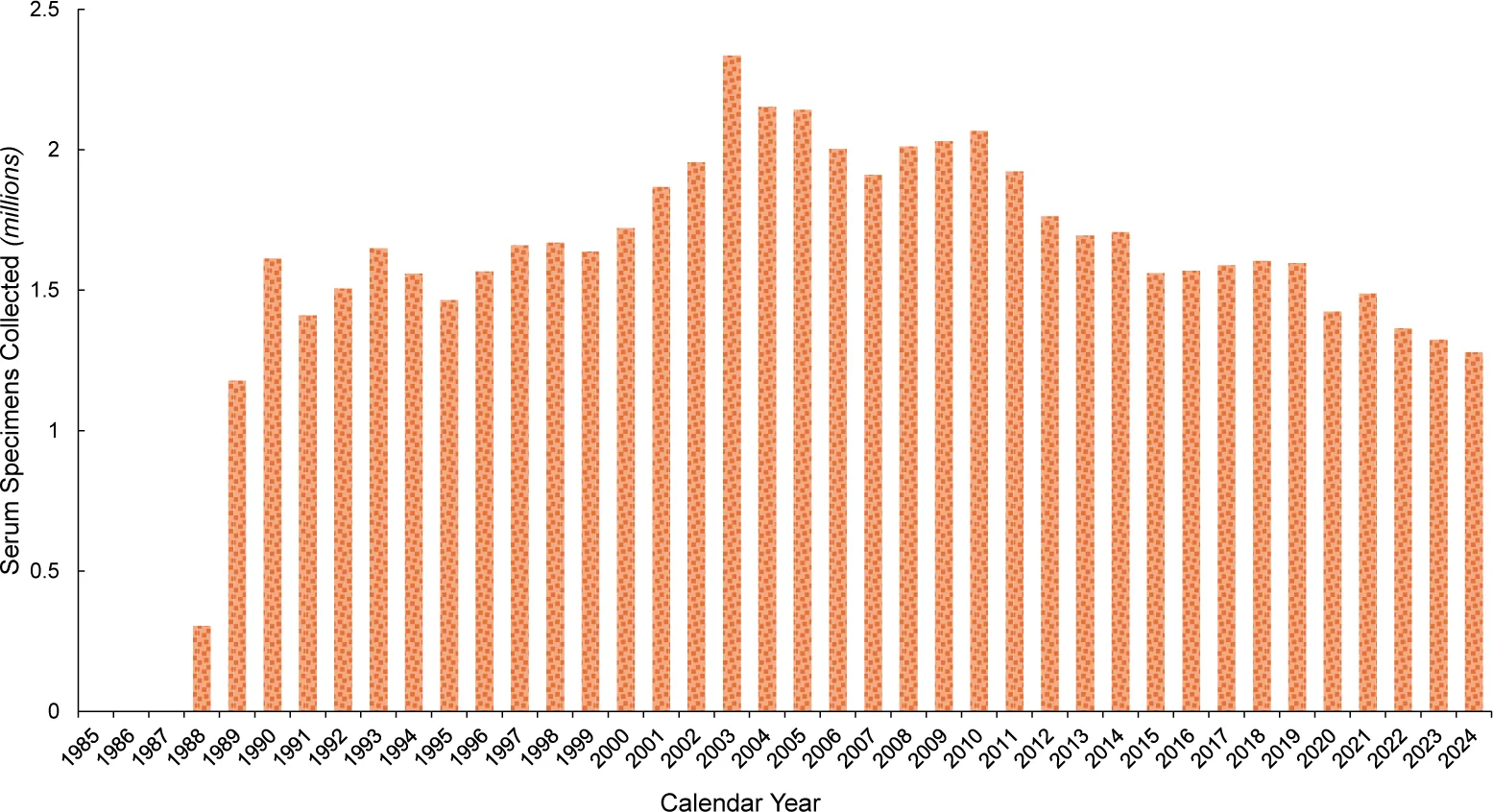
Distribution of Total Department of War Serum Repository Specimens, by Year

**Figure 3** shows the distribution of age at time of serum collection for service members ages 17-70 years. 21-year-olds contributed the highest number of serum samples (n=4.1 million, 6.8%). From 21 years of age, serum contribution decreased with increasing age **(Figure 3)** . Service members who were younger than age 30 years contributed 68.6% of all serum specimens during the surveillance period, while those older than age 50 years contributed only 1.7% of serum samples **(Figure 3)** . The majority (69.3%) of service members provided 3 or more specimens to the DOWSR, with over one-third (36.1%) contributing 6 or more specimens **(Table 2)** .

**FIGURE 3.**
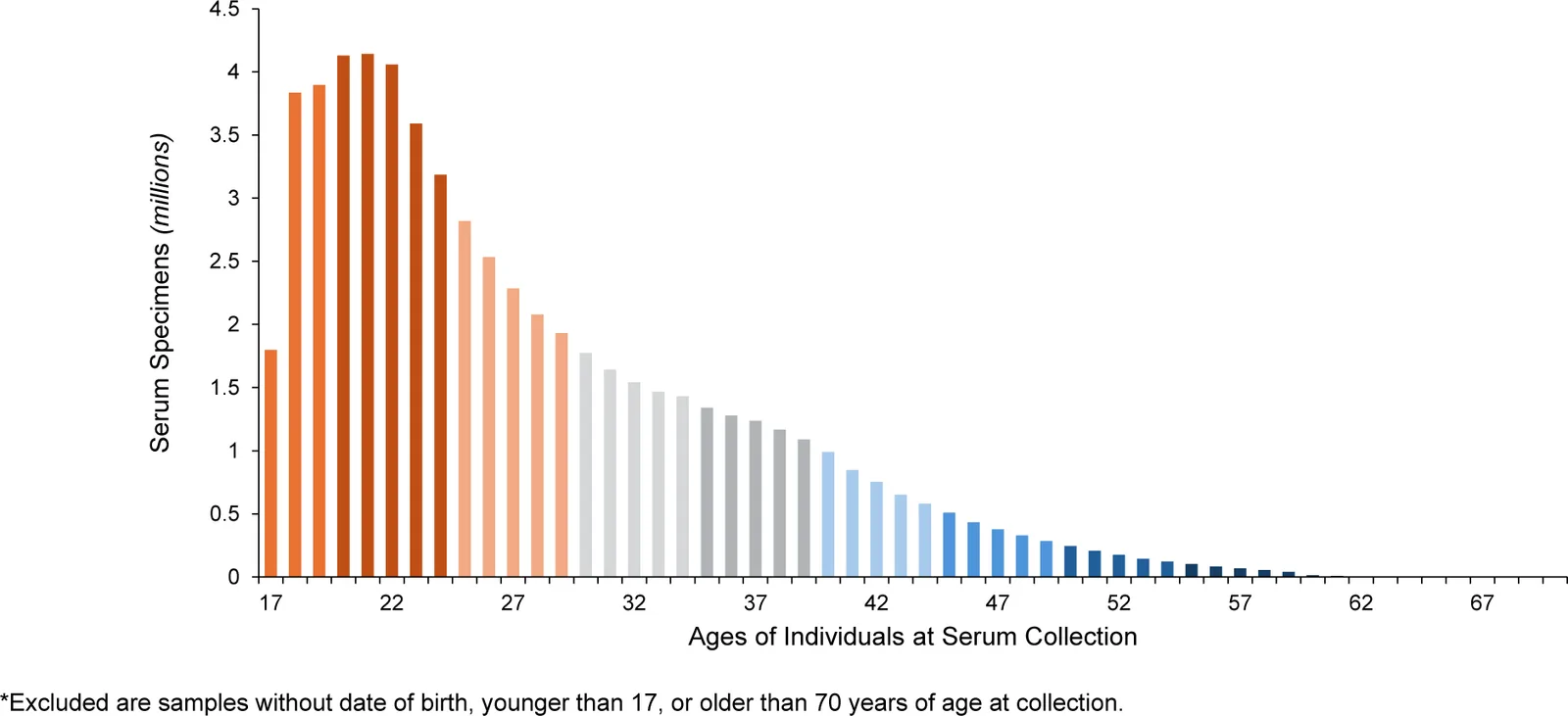
Distribution of Ages at Serum Collections for Department of War Serum Repository ^a^

### Publication of studies using DOWSR specimens, 2013–2024

Combining external and internal search methods **(Figure 1)** , a total of 112 published studies that utilized specimen from the DOWSR were identified; this study determined that 21 of the supported studies resulted in non-peer reviewed reports. Among the 112 studies, 137 analytes were investigated, with some studies investigating more than 1 analyte **(Table 3** , **Table 4)** . Four of the most common analytes studied were regulatory molecules (22 studies), microRNA expression patterns (9 studies), rheumatoid arthritis biomarkers (8 studies), and 25-hydroxyvitamin D (6 studies) **(Table 3)** .

The studies investigating regulatory molecules, which included metabolites, hormones, chemokines, and cytokines, were varied and included studies on numerous cancers, multiple sclerosis, traumatic brain injury, effects of burn pit exposure, lupus, and many more (data not shown). As an example of research conducted using regulatory analytes, 1 study evaluated the association between pentraxin 3 (PTX3) and schizophrenia and bipolar disorder and found that low serum levels of PTX 3 were predictive of schizophrenia. ^9^

Of the 9 studies that analyzed microRNA expression patterns, 5 investigated burn pit deployment outcomes and non-specific environmental exposures. These deployment studies showed that miRNAs were a valid measure for environmental exposures and polycyclic aromatic hydrocarbons (PAH) exposures were elevated post-deployment. ^10 , 11^ The other studies investigating microRNA expression patterns comprised 2 feasibility or pilot studies, 1 methods validation study, and 1 study of miRNA dysregulation due to post-traumatic stress disorder (PTSD). ^12^ Also investigating environmental exposures, studies evaluating persistent organic pollutants known to be endocrine disruptors, such as polybrominated diphenyl ethers (PBDEs), polybrominated biphenyls (PBBs) and polychlorinated biphenyls (PCBs), and per- and polyfluoroalkyl substances (PFAS) showed evidence to suggest that being an Air Force firefighter was a major predictor of measurable PFAS, which was directly linked to higher risk of testicular germ cell tumors **(Table 3)** . ^23^

Of the 8 rheumatoid arthritis studies, 5 investigated anti-cyclic citrullinated peptides (ACCPs) and found evidence both that ACCPs were good predictors of future rheumatoid arthritis diagnoses and were associated with more rapid progression from pre-clinical to clinical rheumatoid arthritis. ^13 - 17^

Studies investigating 25-hydroxyvitamin D (25[OH]D) included a study that showed subjects in the lowest octile of 25(OH)D had the highest risk of suicide. ^18^ An influenza vaccine response study showed no evidence of association between 25(OH)D levels and post-vaccination antibody titers to influenza vaccine. ^19^ The remaining 3 studies, which investigated type 1 diabetes mellitus, Crohn’s disease, and osteoporosis, found a higher risk of type 1 diabetes mellitus among individuals whose 25(OH)D levels were in the lowest 20% of those measured, ^20^ and no significant associations between 25(OH)D levels and Crohn’s disease or osteoporosis, respectively. ^21 , 22^

The most common investigational health outcomes were infectious diseases (30 studies) and autoimmune disorders (25 studies) **(Table 4)** . Neoplasms were the focus of 13 studies, chronic metabolic disorders were the focus of 11 studies, physical injuries were the focus of 7 studies, and burn pit exposures were the focus of 8 studies **(Table 4)** . Four studies were classified as non-specific: 3 were on general environmental exposures, 1 was for the creation of a biore-pository for future study **(Table 4)** .

For comparison of both health exposure and outcome with analytes measured, certain types of analytes were grouped **(Table 4)** : cytokines, chemokines, and hormone-like molecules were grouped generally as regulatory molecules. Studies conducting human DNA sequencing were similarly grouped, and antibodies for specific outcomes were grouped by outcome to simplify the analysis. After analytes were grouped into major categories, 61 studies investigated antibodies, mostly in the infectious disease (n=28) and autoimmune disorder (n=18) categories. Forty-nine studies investigated regulatory molecules and bio-markers, chiefly autoimmune disorders (n=11). Sixteen studies investigated chemical compounds, mostly within the burn pit exposures (n=4) and chronic metabolic (n=4) categories. The 8 studies of nutrients were arrayed among a variety of categories including chronic metabolic (n=2), autoimmune (n=2), and osteoporosis (n=2) studies, and the 3 of human DNA were within infectious disease, autoimmune, and cardiovascular categories **(Table 4)** .

## Discussion

In 1997, the DOWSR began expanding from being chiefly an instrument of the DOW HIV program to support a wider mission of broader health surveillance and identifying, preventing, and controlling service-linked illnesses. ^28 , 30^ It is no surprise that the variety of study topics has continued to increase since inception. The total number of studies and analytes investigated increased from 57 distinct analytes within 76 studies from 1985 to 2012 ^2^ to 65 analytes within 112 studies from 2013 to 2024.

This study noted several differences in the types of analytes and health exposures or outcomes assessed by investigators since the last review of DOWSR-supported publications in 2015. ^2^ In particular, the number of studies analyzing regulatory molecules, miRNAs, and autoantibodies increased. Although the leading investigational outcomes (e.g., infectious disease, autoimmune disorders, neoplasms) were the same in both periods, this study indicates that in the past decade attention within those outcome categories shifted. In addition, studies investigating chronic metabolic conditions, physical injuries, and deployment-related exposures increased.

Reflecting the initial goal of the DOWSR, sexually transmitted infections (including HIV and herpes) had previously been the most popular infectious disease study topic. ^2^ In 2013-2024, focus of infectious disease research shifted to respiratory infections (including influenza and SARS-CoV2) and to a wide array of vectorborne diseases including leishmaniasis and rickettsia **(Table 3)** . Increasingly prevalent studies on arboviruses and vector-borne pathogens may reflect changes in operational deployments in areas with higher risk and spread of vector-borne diseases within the U.S. and abroad.

There was a slight increase in the number of studies published on autoimmune disorders compared to the prior review, with rheumatoid arthritis biomarkers representing a large proportion of these studies (n=3 previously, increased to n=8). ^2^ The DOW Peer Reviewed Medical Research Program has funded arthritis research since 2009, and the Fiscal Year 2024 National Defense Authorization Act established a standalone Arthritis Research Program under the Congressionally Directed Medical Research Programs (CDMRP), which will likely continue to increase the number of these DOWSR-supported biomarker studies. ^32^

As the world’s largest serum repository, and with a chronology exceeding 20 years for many individuals, the DOWSR contains pre-diagnostic data for many individuals who have developed rare cancers. This is a well-suited resource to the risk factor analyses increasingly performed for cancer studies. Increased neoplasm research has likely been fueled by the cancer “moonshot” initiatives of 2016 and 2022, which sought to engage private and public resources and expertise to accelerate development of cancer treatments, diagnostics, and prevention. ^33^ Research projects led by the Murtha Cancer Center ^26^ helped advocate for increased cancer research related to military exposures through the Framingham and Project for Military Exposures and Toxin History Evaluation in US service members (PROMETHEUS) initiatives. The trends seen in this review—an increase from the original review’s 11 studies targeting neoplasms within 27 years to 13 studies within 11 years—correlate with the broader priorities of those initiatives and emphasize the growing utilization of the DOWSR for oncological research.

Studies on burn pit and other environmental or deployment-related exposures became more common following the 2016 publication of the *Journal of Occupational and Environmental Medicine* , ^29^ which highlighted deployment exposures, biomarkers, and health outcomes studies utilizing specimens from the DOWSR; there was only a single burn pit study from 1985 to 2012, compared to 8 from 2013 to 2024. ^2^ Many environmental exposure studies are linked to concerns about exposures at specific bases, such as radioactive, heavy metal, pesticide, and PFAS. These studies have become more common following passage of the Sergeant First Class Heath Robinson Honoring Our Promise to Address Comprehensive Toxics (PACT) Act of 2022, which mandated that the Department of Veterans Affairs (VA) and other federal agencies conduct new health and exposure research studies. ^24^ The PACT Act also helped establish the VA ‘presumption of exposure’ policy, which assumes that veterans who served in specific locations during certain periods of time were exposed to the harmful chemicals used (with Agent Orange an explicit example); this reduces the burden of proof for veterans seeking care. ^24^ Further, the CDMRP has expanded over the past decade, funding research into numerous conditions affecting service members including toxic exposures. ^25^ Research using the presumptive exposure model may become more common in DOWSR studies, with increased veterans’ advocacy to understand exposure associations with health outcomes, and as laboratory methods for metabolomic and genomic studies improve.

The primary limitation of this study is that investigators were not systemically contacted, which means it was possible for some studies to have been missed in this review, particularly studies with ‘negative’ or inconclusive findings, as those are less likely to be published. In addition, abstracts were not included in this review. Because AFHSD tracks all requests for serum as part of its record management systems, it is unlikely that a significant number of published studies are missing from this review, however.

Findings from this review of studies published from 2013 through 2024 indicate that DOW researchers and public health professionals have continued to utilize the DOWSR to enhance military readiness by contributing to the etiological understanding of debilitating conditions, as well as providing important information to improve our understanding of the spread of infectious diseases. The DOWSR is a crucial resource for maintaining a healthy and deployable force. Its importance lies in its ability for researchers and public health personnel to efficiently and retrospectively analyze emergent pathogens, novel exposures, and previously unrecognized health threats to inform risk assessments and validate force health protection measures.
