# Supplementary material for: A New Era of Research in the U.S. Department of War Serum Repository: A Review of Published Studies, 2013–2024
Source: MSMR. 2026 Sep 20;33(9):17–29. (PMC13727151)
Supplement: Supplementary file 1 [file msmr-33-9-17-s01.pdf]

SUPPLEMENTARY TABLE. Studies Included in Analyses Utilizing Department of War Serum Repository Samples, with Categories Assigned, 2013–2024

Title	Journal, Book or Database	Publication Year	Category
Performance of Anti-Cyclic Citrullinated Peptide Assays Differs in Subjects at Increased Risk of Rheumatoid Arthritis and Subjects with Established Disease	*Arthritis & Rheumatology*	2013	Auto-immune disorder or disease
Low Vitamin D Status and Suicide: A Case-Control Study of Active Duty Military Service Members	*PLoS One*	2013	Physical injury
Preclinical Serum 25-Hydroxyvitamin D Levels and Risk of Type 1 Diabetes in a Cohort of US Military Personnel	*American Journal of Epidemiology*	2013	Chronic metabolic
Elevated Subclinical Double-Stranded DNA Antibodies and Future Proliferative Lupus Nephritis	*Clinical Journal of the American Society of Nephrology*	2013	Auto-immune disorder or disease
Relation Between Asymptomatic Proteinase 3 Antibodies and Future Granulomatosis with Polyangiitis	*Clinical Journal of the American Society of Nephrology*	2013	Auto-immune disorder or disease
HPV Seroprevalence Among Servicemembers Entering Military Service During 2011-2012	*MSMR*	2013	Infectious diseases
Increased Serum Free Light Chains Precede the Presentation of Immunoglobulin Light Chain Amyloidosis	*Journal of Clinical Oncology*	2014	Chronic metabolic
Adenovirus 36 Antibodies Associated with Clinical Diagnosis of Overweight/Obesity But Not BMI Gain: A Military Cohort Study	*Journal of Clinical Endocrinology and Metabolism*	2014	Chronic metabolic
Serum Proteomic Analysis of a Pre-Symptomatic Multiple Sclerosis Cohort	*European Journal of Neurology*	2015	Auto-immune disorder or disease
Effects of Traumatic Amputation on β-Trace Protein and β2-Microglobulin Concentrations in Male Soldiers	*American Journal of Nephrology*	2015	Physical injury
Anti-Carbamylated Protein Antibodies Are Present Prior to Rheumatoid Arthritis and Are Associated with its Future Diagnosis	*Journal of Rheumatology*	2015	Auto-immune disorder or disease
Seroconversion to Japanese Encephalitis Virus Among U.S. Infantry Forces in Korea	*American Journal of Tropical Medicine and Hygiene*	2015	Infectious diseases
Seroprevalence and Seroincidence of Herpes simplex Virus (2006-2010), Syphilis (2006-2010), and Vaccine-Preventable Human Papillomavirus Subtypes (2000-2010) Among US Military Personnel	*Sexually Transmitted Diseases*	2015	Infectious diseases
Seroprotective Antibodies to 2011 Variant Influenza A(H3N2v) and Seasonal Influenza A(H3N2) Among Three Age Groups of US Department of Defense Service Members	*PLoS One*	2015	Infectious diseases
Pilot Metabolome-wide Association Study of Benzo(a)pyrene in Serum From Military Personnel	*Journal of Occupational and Environmental Medicine*	2016	Burn pit
Detection of Serum microRNAs from Department of Defense Serum Repository: Correlation With Cotinine, Cytokine, and Polycyclic Aromatic Hydrocarbon Levels	*Journal of Occupational and Environmental Medicine*	2016	Chronic metabolic
High-Resolution Metabolomics for Nutrition and Health Assessment of Armed Forces Personnel	*Journal of Occupational and Environmental Medicine*	2016	Chronic metabolic
High-Resolution Metabolomics Assessment of Military Personnel: Evaluating Analytical Strategies for Chemical Detection	*Journal of Occupational and Environmental Medicine*	2016	Chronic metabolic
Longitudinal Plasma Metanephrines Preceding Pheochromocytoma Diagnosis: A Retrospective Case-Control Serum Repository Study	*European Journal of Endocrinology*	2016	Neoplasm
The Association Between Serum Biomarkers of Collagen Turnover and Subsequent Anterior Cruciate Ligament Rupture	*American Journal of Sports Medicine*	2016	Physical injury
MicroRNA Expression Profiling of the Armed Forces Health Surveillance Branch Cohort for Identification of "Enviro-miRs" Associated with Deployment-Based Environmental Exposure	*Journal of Occupational and Environmental Medicine*	2016	Burn pit
Metabolic Pathways and Networks Associated with Tobacco Use in Military Personnel	*Journal of Occupational and Environmental Medicine*	2016	Tobacco use
Serologic Microbial Associated Markers Can Predict Crohn's Disease Behaviour Years Before Disease Diagnosis	*Alimentary Pharmacology & Therapeutics*	2016	Auto-immune disorder or disease
Altered Type II Interferon Precedes Autoantibody Accrual and Elevated Type I Interferon Activity Prior to Systemic Lupus Erythematosus Classification	*Annals of the Rheumatic Diseases*	2016	Auto-immune disorder or disease
Identification of Extracellular miRNA in Archived Serum Samples by Next-Generation Sequencing from RNA Extracted Using Multiple Methods	*Molecular Biology Reports*	2016	Chronic metabolic

SUPPLEMENTARY TABLE. Studies Included in Analyses Utilizing Department of War Serum Repository Samples, with Categories Assigned, 2013–2024

Title	Journal, Book or Database	Publication Year	Category
Introduction to Department of Defense Research on Burn Pits, Biomarkers, and Health Outcomes Related to Deployment in Iraq and Afghanistan	*Journal of Occupational and Environmental Medicine*	2016	Burn pit
Immune Responses in U.S. Military Personnel Who Received Meningococcal Conjugate Vaccine (MenACWY) Concomitantly with Other Vaccines Were Higher than in Personnel Who Received MenACWY Alone	*Clinical and Vaccine Immunology*	2016	Vaccine-induced immunity
Anti-Myeloperoxidase Antibodies Associate with Future Proliferative Lupus Nephritis	*Autoimmune Diseases*	2017	Auto-immune disorder or disease
Elevated Serum Levels of sCD30 and IL6 and Detectable IL10 Precede Classical Hodgkin Lymphoma Diagnosis	*Cancer Epidemiology, Biomarkers & Prevention*	2017	Neoplasm
Dengue Virus Exposures Among Deployed U.S. Military Personnel	*American Journal of Tropical Medicine and Hygiene*	2017	Infectious diseases
Thyroid-Stimulating Hormone, Thyroid Hormones, and Risk of Papillary Thyroid Cancer: A Nested Case-Control Study	*Cancer Epidemiology, Biomarkers & Prevention*	2017	Neoplasm
Serially Measured Pre-Diagnostic Levels of Serum Cytokines and Risk of Brain Cancer in Active Component Military Personnel	*British Journal of Cancer*	2018	Neoplasm
Combination of Anti-Citrullinated Protein Antibodies and Rheumatoid Factor Is Associated with Increased Systemic Inflammatory Mediators and More Rapid Progression from Preclinical to Clinical Rheumatoid Arthritis	*Clinical Immunology (Orlando)*	2018	Auto-immune disorder or disease
25-Hydroxyvitamin D, Influenza Vaccine Response and Healthcare Encounters Among a Young Adult Population	*PLoS One*	2018	Vaccine-induced immunity
Asymptomatic Visceral *Leishmania infantum* Infection in US Soldiers Deployed to Iraq	*Clinical Infectious Diseases*	2018	Infectious diseases
Metabolome-wide Association Study of Deployment to Balad, Iraq or Bagram, Afghanistan	*Journal of Occupational and Environmental Medicine*	2019	Deployment exposures
Use of Biomarkers to Assess Environmental Exposures and Health Outcomes in Deployed Troops	*Journal of Occupational and Environmental Medicine*	2019	Burn pit
Mumps Outbreak and MMR IgG Surveillance as a Predictor for Immunity in Military Trainees	*Vaccine*	2019	Infectious diseases
Autoantibodies are Present Before the Clinical Diagnosis of Systemic Sclerosis	*PLoS One*	2019	Auto-immune disorder or disease
Cohort Profile of the Proteomic Evaluation and Discovery in an IBD Cohort of Tri-Service Subjects (PREDICTS) Study: Rationale, Organization, Design, and Baseline Characteristics	*Contemporary Clinical Trials Communications*	2019	Non-specific
Machine Learning Approach for Predicting Past Environmental Exposures From Molecular Profiling of Post-Exposure Human Serum Samples	*Journal of Occupational and Environmental Medicine*	2019	Non-specific
Integrative Network Analysis Linking Clinical Outcomes with Environmental Exposures and Molecular Variations in Service Personnel Deployed to Balad and Bagram	*Journal of Occupational and Environmental Medicine*	2019	Non-specific
Neutralizing and Neuraminidase Antibodies Correlate with Protection Against Influenza During a Late Season A/H3N2 Outbreak Among Unvaccinated Military Recruits	*Clinical Infectious Diseases*	2019	Vaccine-induced immunity
Detection of Head and Neck Cancer Based on Longitudinal Changes in Serum Protein Abundance	*Cancer Epidemiology, Biomarkers & Prevention*	2020	Neoplasm
Timing of Elevations of Autoantibody Isotypes Prior to Diagnosis of Rheumatoid Arthritis	*Arthritis & Rheumatology*	2020	Auto-immune disorder or disease
Detection of PLA2R Autoantibodies Before the Diagnosis of Membranous Nephropathy	*Journal of the American Society of Nephrology*	2020	Auto-immune disorder or disease
Serum Neurofilament Light Chain Levels in Patients with Presymptomatic Multiple Sclerosis	*JAMA Neurology*	2020	Auto-immune disorder or disease
Polybrominated Diphenyl Ethers, Polybrominated Biphenyls, and Risk of Papillary Thyroid Cancer: A Nested Case-Control Study	*American Journal of Epidemiology*	2020	Neoplasm
Blast Traumatic Brain Injury and Serum Inflammatory Cytokines: A Repeated Measures Case-Control Study Among U.S. Military Service Members	*Journal of Neuroinflammation*	2020	Physical Injury

SUPPLEMENTARY TABLE. Studies Included in Analyses Utilizing Department of War Serum Repository Samples, with Categories Assigned, 2013–2024

Title	Journal, Book or Database	Publication Year	Category
The Serogroup B Meningococcal Outer Membrane Vesicle-based Vaccine 4CMenB Induces Cross-Species Protection Against *Neisseria gonorrhoeae*	*PLoS Pathogens*	2020	Infectious diseases
Duration of Seropositivity Following Yellow Fever Vaccination in U.S. Military Service Members	*Vaccine*	2020	Infectious diseases
Cohort Profile of a US Military Population for Evaluating Pre-Disease and Disease Serological Biomarkers in Rheumatoid and Reactive Arthritis: Rationale, Organization, Design, and Baseline Characteristics	*Contemporary Clinical Trials Communications*	2020	Auto-immune disorder or disease
Serum Biomarkers Identify Patients Who Will Develop Inflammatory Bowel Diseases up to 5 Years Before Diagnosis	*Gastroenterology*	2020	Chronic metabolic
Leptospirosis Seroconversion Surveillance Among US Marines Assigned to Japan, 2011–2015	*Military Medicine*	2020	Infectious diseases
Levels of Vitamin D Are Low After Crohn's Disease Is Established But Not Before	*Clinical Gastroenterology and Hepatology*	2020	Auto-immune disorder or disease
Circulating Blood Metabolite Trajectories and Risk of Rheumatoid Arthritis Among Military Personnel in the Department of Defense Biorepository	*Annals of the Rheumatic Diseases*	2021	Auto-immune disorder or disease
Combinations of Anticyclic Citrullinated Protein Antibody, Rheumatoid Factor, and Serum Calprotectin Positivity Are Associated with the Diagnosis of Rheumatoid Arthritis Within 3 Years	*ACR Open Rheumatology*	2021	Auto-immune disorder or disease
Serum Fatty Acid Latent Classes Are Associated with Suicide in a Large Military Personnel Sample	*Journal of Clinical Psychiatry*	2021	Physical Injury
Cohort Profile: A Migratory Cohort Study of US Marines Who Train in Australia	*BMJ Open*	2021	Infectious diseases
The Human Immune Response to Saliva of *Phlebotomus alexandri*, the Vector of Visceral Leishmaniasis in Iraq, and Its Relationship to Sand Fly Exposure and Infection	*PLoS Neglected Tropical Diseases*	2021	Infectious diseases
Antibodies to Citrullinated Protein Antigens, Rheumatoid Factor Isotypes and the Shared Epitope and the Near-Term Development of Clinically-Apparent Rheumatoid Arthritis	*Frontiers in Immunology*	2022	Auto-immune disorder or disease
The Seroepidemiology of Dengue in a US Military Population Based in Puerto Rico During the Early Phase of the Zika Pandemic	*PLoS Neglected Tropical Diseases*	2022	Infectious diseases
Immune Interference Revisited: Impact of Live-Attenuated Influenza Vaccine Prior to Yellow Fever Vaccination	*Vaccine*	2022	Vaccine-induced immunity
Coccidioidomycosis Seroincidence and Risk Among Military Personnel, Naval Air Station Lemoore, San Joaquin Valley, California, USA	*Emerging Infectious Diseases*	2022	Infectious diseases
Pre-Existing Thyroid Autoimmunity and Risk of Papillary Thyroid Cancer: A Nested Case-Control Study of US Active-Duty Personnel	*Journal of Clinical Oncology*	2022	Neoplasm
Antibody Responses to *Phlebotomus papatasi* Saliva in American Soldiers with Cutaneous Leishmaniasis Versus Controls	*Frontiers in Tropical Diseases*	2022	Infectious diseases
Prediagnostic Appearance of Thrombospondin Type-1 Domain 7A Autoantibodies in Membranous Nephropathy	*Kidney360*	2023	Auto-immune disorder or disease
A Predictive Autoantibody Signature in Multiple Sclerosis	*medRxiv.org*	2023	Auto-immune disorder or disease
Use of Longitudinal Serum Analysis and Machine Learning to Develop a Classifier for Cancer Early Detection	*Methods in Molecular Biology*	2023	Neoplasm
Serum Antibodies to Periodontal Pathogens Prior to Rheumatoid Arthritis Diagnosis: A Case-Control Study	*Seminars in Arthritis and Rheumatism*	2023	Auto-immune disorder or disease
Seroprevalence as an Indicator of Undercounting of COVID-19 Cases in a Large Well-Described Cohort	*AJPM Focus*	2023	Infectious diseases
Longitudinal Changes in Immune Activation Serum Biomarkers Prior to Diagnosis and Risk of B-Cell NHL Subtypes	*Cancer Epidemiology, Biomarkers & Prevention*	2023	Neoplasm
A Nested Case-Control Study of Serum Per- and Polyfluoroalkyl Substances and Testicular Germ Cell Tumors among U.S. Air Force Servicemen	*Environmental Health Perspectives*	2023	Neoplasm
Serologic Response to the Epstein-Barr Virus Peptidome and the Risk for Multiple Sclerosis	*JAMA Neurology*	2024	Auto-immune disorder or disease

SUPPLEMENTARY TABLE. Studies Included in Analyses Utilizing Department of War Serum Repository Samples, with Categories Assigned, 2013–2024

Title	Journal, Book or Database	Publication Year	Category
Organochlorine Pesticides and Risk of Papillary Thyroid Cancer in U.S. Military Personnel: A Nested Case-Control Study	*Environmental Health*	2024	Neoplasm
Multi-Autoantibody Testing Identifies Expansion of Reactivity to Targeted Antigens Before a Diagnosis of Rheumatoid Arthritis	*ACR Open Rheumatology*	2024	Auto-immune disorder or disease
Incidence of Alpha-Gal IgE Sensitization in 3000 Military Personnel, Assessing Sex, Race, Installation, and Occupational Impacts	*Journal of Clinical Medicine*	2024	Infectious diseases
Smoking and Multiple Sclerosis Risk in Black People: A Nested Case-Control Study	*Multiple Sclerosis and Related Disorders*	2024	Auto-immune disorder or disease
Changes in the Seroprevalence of Tick-Borne Rickettsia and Ehrlichia Among Soldiers: Fort Liberty, North Carolina, 1991–2019	*Journal of Infectious Diseases*	2024	Infectious diseases
Identifying a Level of Neutralizing Antibody that Correlates with Protection from Clinical Mumps Disease During a 2017 Mumps Outbreak Among Military Service Members	*Open Forum Infectious Diseases*	2024	Infectious diseases
Development of Automated Microfluidic Immunoassays for the Detection of SARS-CoV-2 Antibodies and Antigen	*Journal of Immunological Methods*	2024	Infectious diseases
Serum Concentrations of Persistent Endocrine-Disrupting Chemicals in U.S. Military Personnel: A Comparison by Race/Ethnicity and Sex	*International Journal of Hygiene and Environmental Health*	2025	Chronic metabolic
Serum Proteomic Signatures Before the Diagnosis of Rheumatoid Arthritis: Evolving Biologic Pathways and Specific Periods of Disease Development	*Arthritis & Rheumatology*	2025	Auto-immune disorder or disease
Circulating Inflammation Biomarkers and the Risk of Esophageal Adenocarcinoma: A Nested Case-Control Study in the Department of Defense Serum Repository	*Cancer Epidemiology, Biomarkers & Prevention*	2025	Neoplasm
Long-Term miRNA Changes Predicting Resiliency Factors of Post-Traumatic Stress Disorder in a Large Military Cohort–Millennium Cohort Study	*International Journal of Molecular Sciences*	2025	Mental illness
Post-Vaccination Serum Cytokines Levels Correlate with Breakthrough Influenza Infections	*Scientific Reports*	2023	Infectious diseases
Leptospirosis and Rickettsial Diseases Sero-Conversion Surveillance Among U.S. Military Personnel in Honduras	*Military Medicine*	2022	Infectious diseases
Changes in Serum Biomarkers of Cartilage Turnover After Anterior Cruciate Ligament Injury	*American Journal of Sports Medicine*	2013	Physical injury
Early Screening and Diagnosis for Low Bone Mineral Density Utilizing Opportunistic Screening, Serum Biomarkers, and Advanced Modeling	DTIC	2023	Osteoporosis
Integrative Cardiac Health Project	DTIC	2019	Cardiovascular disease
Vitamin D Supplementation for Prevention of Post-Traumatic Osteoarthritis: Evaluation in Animal and Clinical Models	DTIC	2019	Osteoporosis
Persistence of Serogroup C Antibody Responses Following Quadrivalent Meningococcal Conjugate Vaccination in United States Military Personnel	*Vaccine*	2014	Infectious diseases
Blast Concussion mTBI, Hypopituitarism, and Psychological Health in OIF/OEFVeterans	DTIC	2014	Physical injury
Infectious Related Triggers of Crohn's Disease in Susceptible Military Personnel		2015	Chronic metabolic
GlycomePREDICTS: IgG Glycome as a Predictor of Inflammatory Bowel Disease Development	DTIC	2019	Chronic metabolic
Humoral Immunity to Primary Smallpox Vaccination: Impact of Childhood Versus Adult Immunization on Vaccinia Vector Vaccine Development in Military Populations.		2017	Vaccine-induced immunity
Predictors of the Onset of Schizophrenia in US Military Personnel	*Journal of Nervous and Mental Disease*	2015	Mental illness
Assessment of Lyme Seroconversion Among US Military Personnel in Honduras	*Military Medicine*	2025	Infectious diseases
Hepatitis B Seroprevalence in the U.S. Military and Its Impact on Potential Screening Strategies	*Military Medicine*	2020	Infectious diseases
Different Repeat Annual Influenza Vaccinations Improve the Antibody Response to Drifted Influenza Strains	*Scientific Reports*	2017	Vaccine-induced immunity
Costs and Consequences: Hepatitis C Seroprevalence in the Military and Its Impact on Potential Screening Strategies	*Hepatology*	2016	Infectious diseases

SUPPLEMENTARY TABLE. Studies Included in Analyses Utilizing Department of War Serum Repository Samples, with Categories Assigned, 2013–2024

Title	Journal, Book or Database	Publication Year	Category
Low Risk for Ross River Virus Infection in Expeditionary Forces Training in Australia Demonstrated by a Serological Survey of Marine Rotational Force-Darwin, 2012–2018	*MSMR*	2023	Infectious diseases
Monocyte Activation Detected Prior to a Diagnosis of Schizophrenia in the US Military New Onset Psychosis Project (MNOPP)	*Journal of Schizophrenia Research*	2018	Mental illness
The Deepwater Horizon Oil Spill Coast Guard Cohort Study	*Occupational and Environmental Medicine*	2018	Non-specific
Increased Risk of Monoclonal Gammopathy of Undetermined Significance in US Military Service Members: A Case-Control Study of 1,068 Service Members Deployed to Either Europe or Iraq, with or Without Reported Burn Pit and Toxic Smoke Exposure	*Blood*	2023	Non-specific
Longitudinal Analysis of Circulating Markers of Bone Turnover Across Multiple Decades in Osteoporotic Women	*Journal of Hand Surgery (American Vol.)*	2022	Osteoporosis
Live Oral Adenovirus Type 4 and Type 7 Vaccine Induces Durable Antibody Response	*Vaccines (Basel)*	2020	Infectious diseases
Analysis of Postdeployment Serum Samples Identifies Potential Biomarkers of Exposure to Burn Pits and Other Environmental Hazards	*Journal of Occupational and Environmental Medicine*	2019	Non-specific
A Nested Case-Control Study of Serum Polychlorinated Biphenyls and Papillary Thyroid Cancer Risk Among U.S. Military Service Members	*Environmental Research*	2022	Neoplasm
Assessing Health Outcomes After Environmental Exposures Associated with Open Pit Burning in Deployed US Service Members	*Journal of Occupational and Environmental Medicine*	2016	Non-specific
MicroRNAs as Novel Biomarkers of Deployment Status and Exposure to Polychlorinated Dibenzo-p-Dioxins/Dibenzofurans	*Journal of Occupational and Environmental Medicine*	2016	Deployment exposures
Polycyclic Aromatic Hydrocarbons and Polychlorinated Dibenzo-p-Dioxins/Dibenzofurans in Microliter Samples of Human Serum as Exposure Indicators	*Journal of Occupational and Environmental Medicine*	2016	Non-specific
